# Control Efficacy and Deposition Characteristics of an Unmanned Aerial Spray System Low-Volume Application on Corn Fall Armyworm *Spodoptera frugiperda*

**DOI:** 10.3389/fpls.2022.900939

**Published:** 2022-09-13

**Authors:** Changfeng Shan, Jiajun Wu, Cancan Song, Shengde Chen, Juan Wang, Haihong Wang, Guobin Wang, Yubin Lan

**Affiliations:** ^1^College of Agricultural Engineering and Food Science, Shandong University of Technology, Zibo, China; ^2^Corteva Agroscience Technology (Shanghai) Co., Ltd., Shanghai, China; ^3^College of Electronic Engineering, College of Artificial Intelligence, South China Agricultural University, Guangzhou, China; ^4^Mechanical and Electrical Engineering College, Hainan University, Haikou, China; ^5^Department of Biological and Agricultural Engineering, Texas A&M University, College Station, TX, United States

**Keywords:** unmanned aerial spray system, spray volume, droplet deposition, control efficacy, plant protection

## Abstract

As a major global pest, fall armyworm (FAW), *Spodoptera frugiperda*, invaded China in 2019, which has seriously threatened the safety of China's food production and raised widespread concerns. As a new low-volume application technology, an unmanned aerial spray system (UASS) is playing an important role in the control of FAW in China. However, the studies on the effect of the water application volume on the efficacy of FAW using UASS have been limited. In this study, Kromekote® cards were used to sample the deposition. The method of using a sampling pole and sampling leaf for the determination of deposition. Four water application volumes (7.5, 15.0, 22.5, and 30.0 L/ha) were evaluated with regard to the corn FAW control efficacy. A blank control was used as a comparison. The control efficacy was assessed at 1, 3, 7, and 14 days after treatment (DAT). The tested results showed that sampling methods have a significant effect on deposition results. The number of spray deposits and coverage on the sampling pole were 35 and 40% higher than those on the sampling leaves, respectively. The deposition and control efficacy gradually increased as the water application volume increased. The control efficacy at 14 DAT under different water application volumes was in the range of 59.4–85.4%. These data suggest that UASS spraying can be used to achieve a satisfying control of FAW, but the control efficacy of the water application volume of 30.0 and 22.5 L/ha did not differ significantly. Considering work efficiency, a water application volume of 22.5 L/ha is recommended for field operation.

## Introduction

The fall armyworm (FAW), *Spodoptera frugiperda*, is a major global pest. The FAW displays outstanding adaptable and migratory capacity and is an agricultural pest characterized by outbreaks. When the temperature is suitable, FAW can lay eggs once every 2–3 days, about 1,500 eggs are laid at a time, and a life cycle can be completed in 30–45 days (Cui et al., 2019). It can travel a 1,600 km migration distance within 30 h if the weather conditions are suitable (Lu et al., 2021). It originated in North America and invaded Africa in 2016. In 2 years, it spread across 44 countries in Africa and caused great damage to corn cultivation (Goergen et al., 2016). Since 2018, FAW has been expanding northward and southward into Asia, and the degree of occurrence has seriously increased, with it now has spread to 16 countries in Asia. In January 2019, it invaded Yunnan Province, China. Since then, it has seriously threatened the safety of China's food production. After 6 months, it was found in more than 22 provinces, seriously threatening the grain production (Jing et al., 2019). It has attracted wide attention internationally due to its strong adaptability, migration ability (Westbrook et al., 2015), and the characteristics of outbreak damage (Johnson, 1987). FAW larvae attack a large number of cultivated plant species (Casmuz et al., 2010), such as corn, sorghum, cotton, peanut, and soybean. In sub-Saharan Africa, more than $13 billion a year is at risk of crops being destroyed by the FAW (Harrison et al., 2019). In the United States, an outbreak year can cost as much as $500 billion in yield loss (Mitchell, 1979; Montezano et al., 2018). In Brazil, about $600 million was spent in 2009 to control FAW (Ferreira Filho et al., 2010). When 55–100% of corn plants were infected with FAW in the mid-to-late corn stage, the yield decreased by 15–73% (Hruska and Gould, 1997). Currently, FAW control is primarily achieved by spraying insecticides with large volume sprays. A crop protection unmanned aerial spray system (UASS) represents a new pesticide spraying technology adapted to the development of modern agriculture. UASS has many advantages compared with manned aircraft and traditional application machinery, including high efficiency, low drift, no need to take off from an airport, a lower price and labor operation cost, and no damage to the physical structure of crops and soil (Zhang et al., 2015). Meanwhile, it is more suitable for complex and tall crops where no machine can normally move. It can fly quickly to the exact location to accurately process the target area, and be pre-programmed to navigate its way around. Furthermore, the use of a low or ultra-low spray volume can reduce pesticide use by 15.0–20.0%, which can be used as an important technical support for the pesticide reduction program in China (Lan and Chen, 2018; Meng et al., 2019).

In recent years, the use of and research on UASS have rapidly developed across the world (Huang et al., 2013; Berner and Chojnacki, 2017). In the most recent 5 years, research on UASS has been carried out in China, the United States, Brazil, Poland, and other countries (Faiçal et al., 2014, 2017; Pachuta et al., 2018). Researchers have studied the addition of additives (He et al., 2017; Xiao et al., 2019), droplet deposition (Qin et al., 2016; He et al., 2017; Zhang et al., 2017; Wang et al., 2019a), control efficacy (Qin et al., 2016; Zhang et al., 2017; Wang et al., 2019a; Xiao et al., 2019), etc., in UASS. Xiao et al. (2019) studied the effects of aviation spray adjuvants on cotton defoliation and boll opening. The results showed that adding aviation spray adjuvants could increase the defoliation rate by 3.1–34.6% and the bell opening rate by 6.7–29.6%. He et al. (2017) studies showed that increasing the water application volume can significantly increase the deposition density of droplets while adding spray adjuvants can significantly increase the deposition and effective deposition rate of droplets. Wang et al. (2019a) studied the effect of a low water application volume on droplet deposition and control efficacy, and the results indicated that different water application volumes significantly influenced the droplet deposition and control efficacy of wheat pests and diseases. Qin et al. (2016) found that flight parameters not only affect the control efficacy of rice planthoppers (*Nilaparvata lugens*) but also affect the droplet distribution uniformity in a rice canopy. Zhang et al. (2017) used UASS to study the effect of different citrus tree shapes on droplet deposition and control efficacy. The results showed that the droplet distribution performance and control efficacy of hedgerow-shaped plants were the best. Xin et al.'s (2018) research showed that with the increase of the UASS water application volume, the thidiazuron and diuron residues in cotton leaves also increased. Phani et al. (2021) studied the effects of different pesticides on the control effect of FAW using high-volume spraying, and screened out the pesticides with better control effect. Yan et al. (2021) studied the control effect of FAW by using a plant protection UASS to spray solid particles of pesticides. Lu et al. (2021) used a plant protection UASS to study the effect of spraying time on the control effect of FAW and recommend the best spraying time. Different application parameters have a great effect on the control of different pests and diseases by plant protection UASS. Meanwhile, in the prevention and control of pests and diseases, excessive water application volume will not only cause the loss of pesticides but also reduce work efficiency, while too low water application volume often fails to achieve the effect of pest control. However, none of the above studies involved the effect of different water application volumes of plant protection UASS on the control effect of FAW. It is unknown whether the plant protection UASS low water consumption spray can effectively control the FAW. Therefore, a crop protection UASS was used to study the effects of four different water application volumes on the control efficacy of FAW, and determine the optimal water application volume.

In the dose transfer process, the deposition structure plays an important role. This is because it associates the target organism with the pesticide application (Ebert et al., 1999). The deposit structure has a significant effect on the control of pests and diseases. However, different sampling materials and sampling arrangement methods have been used to obtain different deposition results. Therefore, the choice of sampling material and sampling arrangement method is very important for the deposition results. Commonly used sampling materials include Kromekote® cards, water-sensitive paper, Petri dishes, and filter paper (Brain et al., 2017). The most common sampling methods include the arrangement of sampling materials on a slant on the plant leaf (Qin et al., 2016; Wang et al., 2019b) or horizontally on the sampling pole (Kharim et al., 2019; Wang et al., 2019a). However, different sampling methods lead to different deposition results, which makes it difficult to compare the data in different papers. Therefore, this study compared the droplet deposition using different sampling methods under the same spray conditions.

An experiment was carried out in Yunnan Province, China to study the control efficacy of UASS on FAW. Due to the climate characteristics of this experiment site, the air humidity is relatively high, and the water-sensitive paper is easily affected by moisture, which can easily affect the test results. The use of stainless steel samplers is more troublesome for subsequent acquisition of test data (droplet density and coverage), while Kromekote cards are similar to water-sensitive paper, which is not easily disturbed by external conditions and has a better stability. Therefore, the experiment chose the Kromekote® card as the deposition acquisition material, and DepositScan was used to obtain the droplet density and coverage. The experiment compared the effect of four different water application volumes (7.5–30.0 L/ha) on the control efficacy of FAW in corn.

## Materials and Methods

### Sprayers

**Figure 1:**
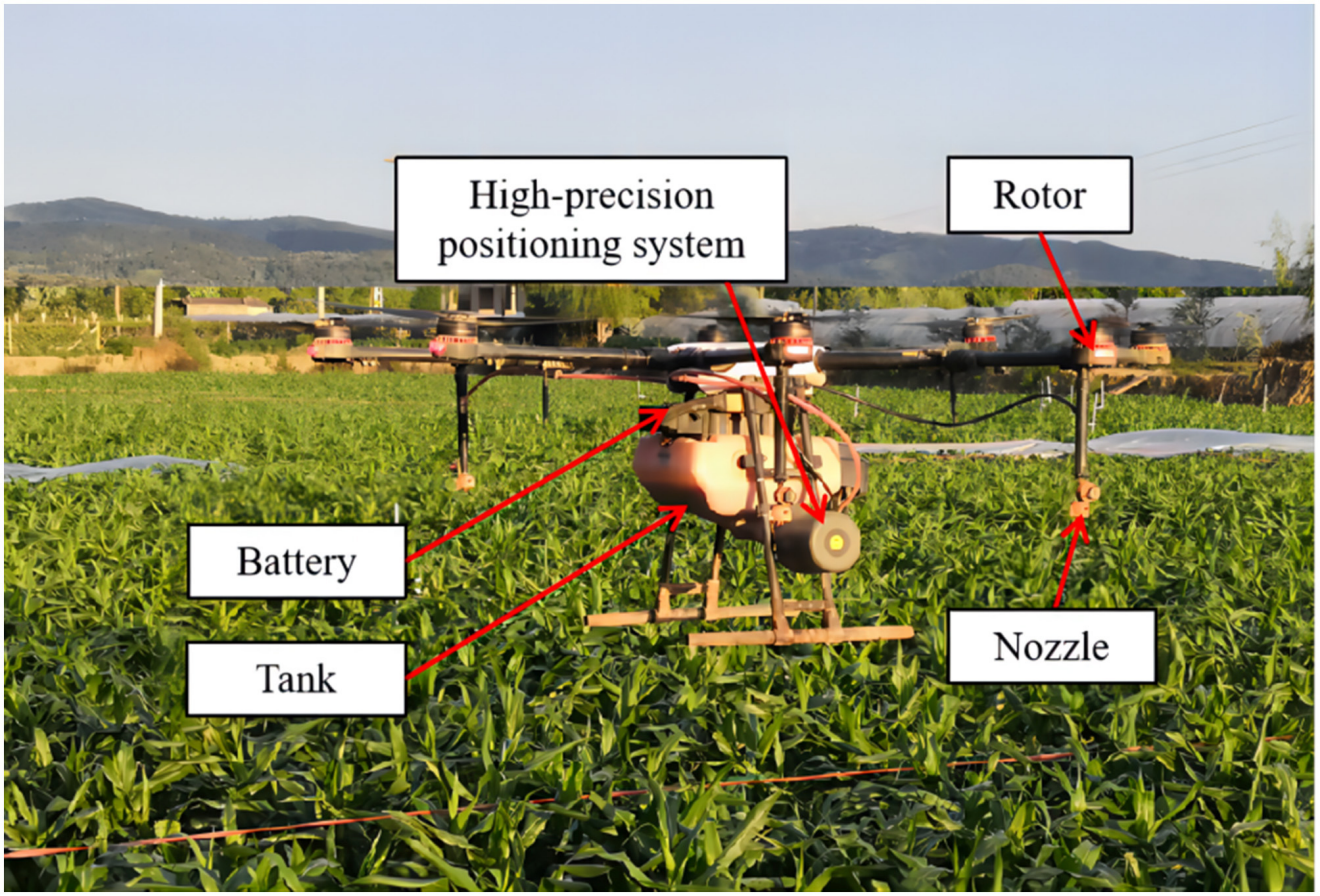
The DJI MG-1P eight-rotor electric unmanned aerial spray system (UASS).

The spraying equipment is an eight-rotor electrical-powered UASS (MG-1P, Shenzhen DJI Technology Co., Ltd., Guangzhou, China). The spraying equipment is shown in Figure 1. The MG-1P UASS is powered by lithium-ion batteries, which provide a flight time of about 15 min on one charge. It can be operated remotely or automatically and can fly according to a pre-programmed route. The MG-1P platform was equipped with four XR11001 or XR110015 nozzles (TeeJet Technologies, Wheaton, IL). Due to the limited range of the UASS flight speed, the water application volumes in this test were difficult to achieve when only using a change in flight speed. Therefore, the tests used XR11001 and XR110015 nozzles to achieve different water application volumes. The nozzles were mounted under rotors and angled vertically downward and in a parallel direction with reference to the direction of flight. The arrangement of the four nozzles was rectangular, and the length and width were 132 and 56 cm, respectively. The spray pressure, output rate, and flight height of the UASS were set through the remote controller. When using XR11001 or XR110015 nozzles, the spray pressure and output rate were 2.0 bar and 0.32 L/min or 2.5 bar and 0.54 L/min, respectively.

### Experimental Design

The experiment was conducted in September 2019 at Corteva Yunnan research center, Kunming City, Yunnan Province (E103°8′52″; N24°46′47″), China (the field is private land, and the owner of the land permitted to conduct the study on this site). The corn variety in the experimental site field was Tian Cui 311, and the sowing time was July 20, 2019. The corn plant height, row spacing, and plant spacing during application were about 0.4, 0.4, and 0.3 m, respectively. The corn growth periods were small, with flaring open stages. FAW kills the growing point of corn plants, causing numerous holes in the whorls and upper leaves (Yan et al., 2021). The field observation and survey rate of the harmed corn plants reached above 10%, and most of the corn plants reached the damage level of 3 (Davis scale) (Davis et al., 1992).

The dimension of the experimental field was about 170 m × 118 m, and it was divided into five treatments. Each treatment was replicated three times for a total of 12 plots. Each plot was a 50 m × 22 m area. Then, 10 m buffer zones between plots were set to avoid the drift pollution of droplets. Among them, there were four treatments for the DJI MG-1P UASS and one treatment for the blank control. In the experiment, the effect of water application volumes on the spray deposition and control effect was studied. The DJI MG-1P UASS used four different water application volumes of 7.5, 15.0, 22.5, and 30.0 L/ha.

#### Sampling Point Arrangement

**Figure 2:**
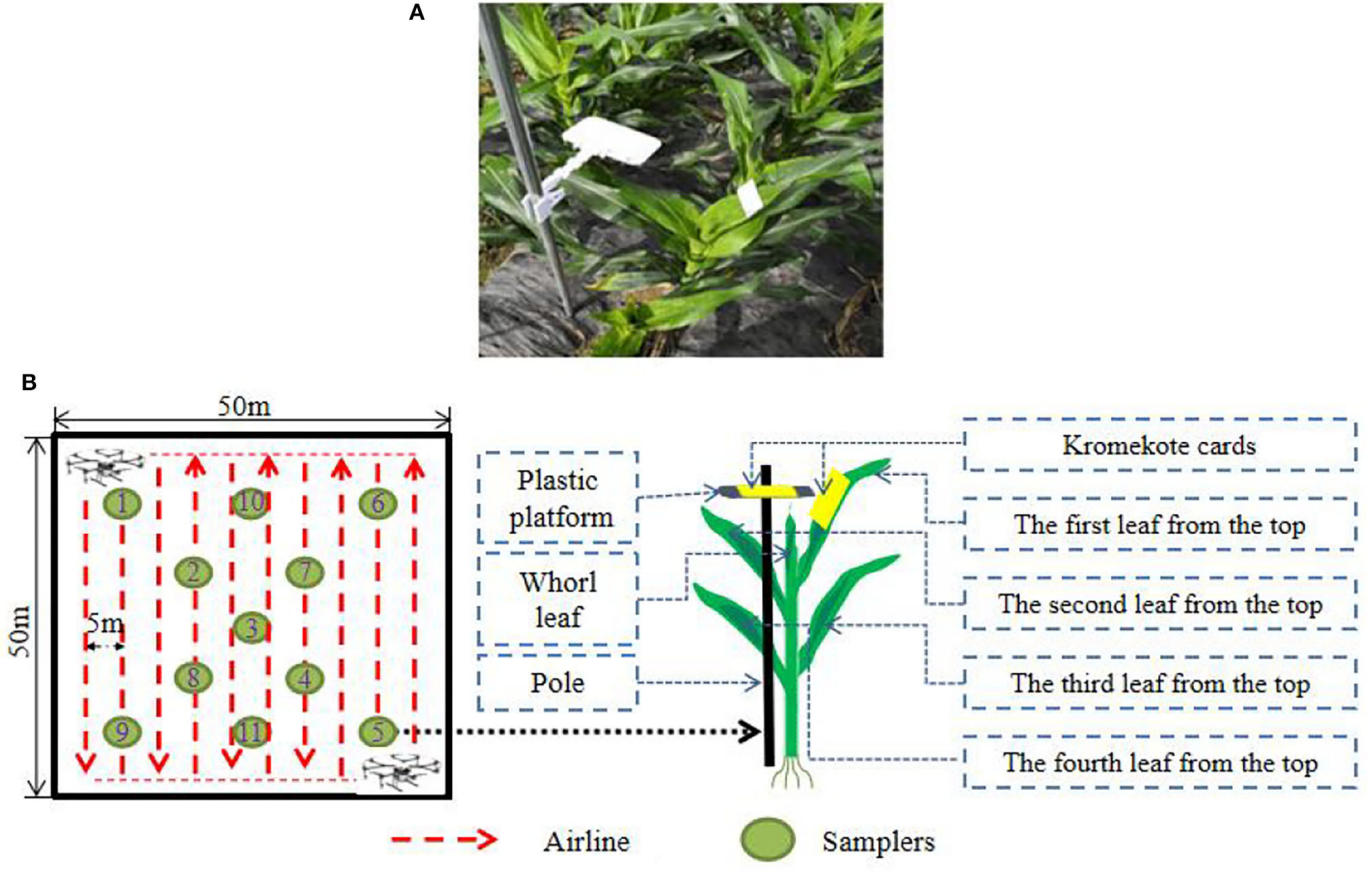
**(A)** The actual arrangement in the field. **(B)** Sampling point arrangement.

Two sampling methods were used to analyze the influence of different sampling methods on the deposition results (Figure 2). The first sampling method was Kromekote® cards horizontally arranged at a distance of 5 cm from the crop canopy using a sampling pole. This method has the characteristic that the deposition sampling efficiency is not related to the crop canopy. This standard method can be used to compare the results with other research in further work. The second sampling method was Kromekote® cards arranged on the first corn leaf from the top with a stapler at an angle of almost 50 ± 10 degrees. The droplet deposition on the first leaf from the top of the corn had an important role in the control efficacy of FAW. There were two main considerations in using this sampling method. On the one hand, the droplet was mainly deposited on the first leaf from the top of the corn; on the other hand, the FAW mainly lays eggs and hatches on the first leaf from the top of the corn (Yan et al., 2021). For analyzing the droplet deposition, 11 sampling points were uniformly arranged in the experiment plot.

#### Water Application Volume

In the experiment, the spray height was 2.0 m. Under the spray pressure of 2.0 and 2.5 bar, the droplet size of the XR11001 nozzle and XR110015 nozzle was 90.4–121.2 μm (Jeon and Tian, 2010) and 154.2–183.0 μm (Guo et al., 2020), respectively. Different water application volumes of UASS were achieved by changing the flight speed and nozzles. The corresponding flight speed according to the water application volume was ascertained under the conditions of spray pressure, output rate, and swath width. The flight speed was calculated according to Formula (1) (American Society of Agricultural Engineers, 1995). When the water application volumes were 7.5, 15.0, 22.5, and 30.0 L/ha, the corresponding flight speeds were as shown in Table 1.


(1) $$\begin{array}{l} V=\frac{K_{3}\;\times \;Q}{RS} \end{array}$$

where, R is the water application volume, L/ha; Q is the output rate, L/min; K_3_ is a constant, 600; V is the flight speed, km/h; and S is the swath width, m.

**Table 1:** The speed of flight under different water application volumes.

**Nozzles**	**Water application volume (L/ha)**	**Spray pressure** **(bar)**	**Droplet size (μm)**	**Output rate** **(L/min)**	**Swath width (m)**	**Flight speed** **(m/s)**
XR11001	7.5	2.0	90.4–121.2	0.32	5.0	5.7
XR110015	15.0	2.5	154.2–183.0	0.54		4.8
	22.5					3.2
	30.0					2.4

### Measurement of Droplet Deposition

Before application, 10.0 g/L of Allura Red (80% purity, purchased from Beijing Oriental Care Trading Ltd., China) was added to the tank using a tracer. The tracer is used to measure the deposition of droplets on Kromekote® cards (Qin et al., 2018). After application, the Kromekote® cards contained in a self-sealing bag were brought to the laboratory for collection and processing. Kromekote® cards were scanned at a resolution of 600 dpi with a scanner (Model GT-1500 Seiko Epson Corporation. Japan). Then, the imagery software DepositScan (USDA, Wooster, OH, USA) was utilized to extract and analyze the droplet density and coverage on the scanned photos (Xiao et al., 2019).

The climatic conditions were recorded using a weather meter (Model NK-5500, Nielsen-Kellerman Co., Boothwyn, PA, 209 USA), which indicated temperatures of 22.9–29.5°C, relative humidity of 45.4–72.2%, and wind velocities of 0.4–2.2 m/s during the deposition test.

### Control Efficacy

The insecticide used in this experiment was a 25% Spinetoram water-dispersible granule (Delegate®) produced by Corteva™ agriscience Company, USA. The dosage for each treatment was 30 g a.i/ha.

**Figure 3:**
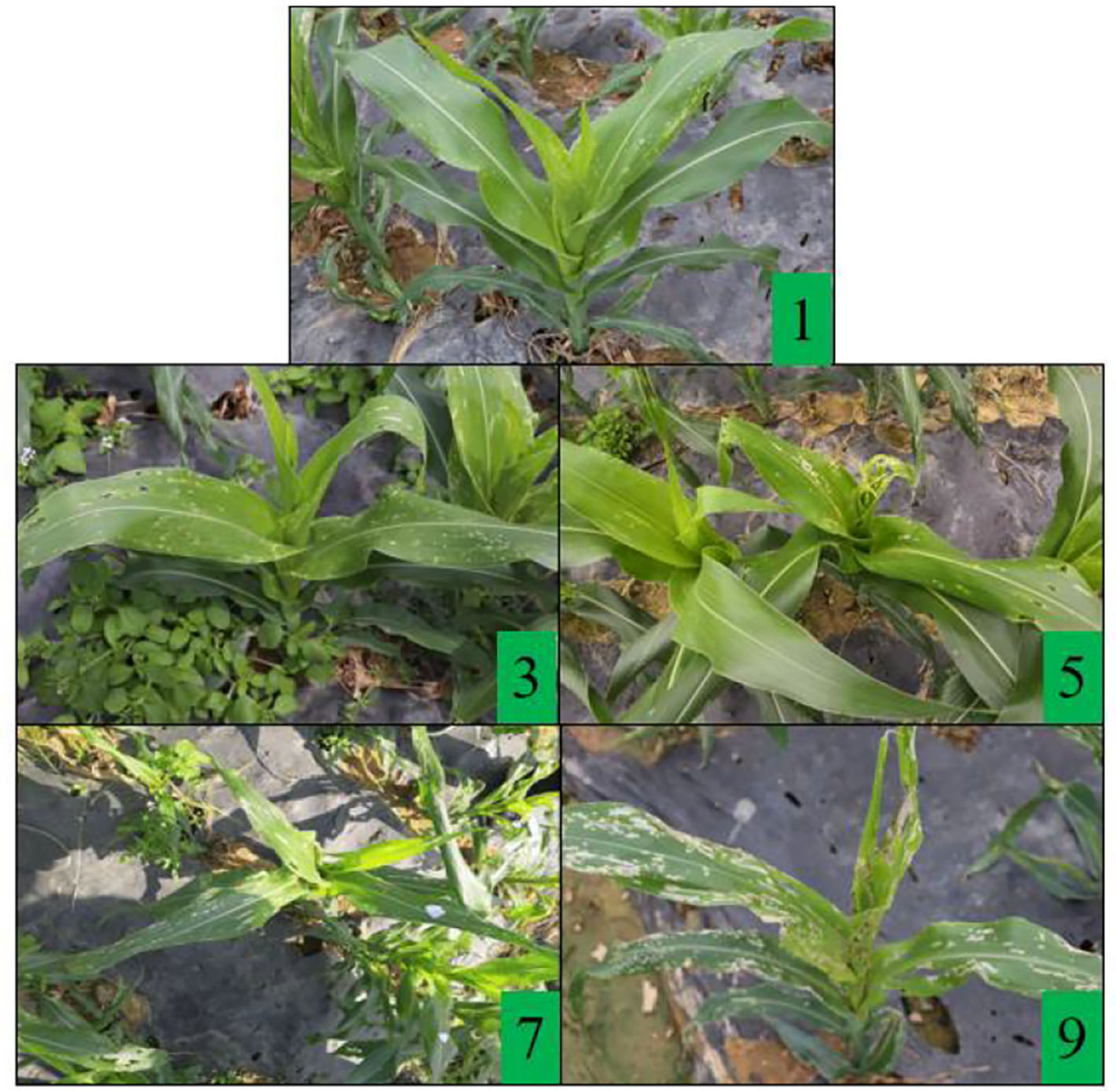
A visual map of some different damage levels of corn fall armyworm. (1) There was only needle-like damage on the leaves, and the damaged area was <5%. (3) There were pin-eye or small annular lesions on the leaves, and the damaged leaf area is between 6 and 15%. (5) Several small and medium irregular holes appeared on the leaves, and the damaged area was between 16 and 25%. (7) There are many large, elongated lesions on the leaves, ranging from 26 to 50% of the damaged area. (9) Corn leaves are destroyed and it is difficult to restore normal growth.

The efficacy experiment was based on the insecticide field efficacy test guideline (II) standards and the Davis scale. A five-point sampling method per plot was selected. The FAW numbers and the damage index of three plants of corn per point before spraying were investigated and the corns were marked with a red string (Wang et al., 2019a,b). Then, 1, 3, 7, and 14 days after application, the number of FAW and the damage index of corn in the same location and plant were investigated again. The overall control efficacy against corn FAW was calculated without regard to the instars of the corn FAW. The control efficacy was obtained based on the population numbers of live insects in each zone before and after spraying. The control efficacy was calculated according to Equations (2) and (3) (Wang et al., 2019b). The damage index of the corn method referred to the investigation method of Davis et al. (1992). Figure 3 shows a visual map of the corn FAW damage to leaf feeding. A numerical scale (0–9) was employed, where 0 indicates no visible damage and 9 indicates heavy damage, which is also known as the Davis scale. This method can quickly and easily distinguish small differences in plant damage. It was based on the types and numbers of feeding lesions at 7 and 14 days after infestation. The damage index of each treatment area was calculated according to the damage index Equation (4).


(2) $$\begin{array}{l} \text{Mortality }(\%)=(\text{The number of pests before application }\\ \;-\text{The number of pests after application})\text{/}\\ \text{ The number of pests before application }\times \text{ 100} \end{array}$$

(3) $$\begin{array}{l} \text{Control effect }(\%)\text{ = }[\text{Observed mortality }(\%)\\ \;-\text{Control mortality }(\%)]\\ \text{ / }[\text{100 -Control mortality }(\%)]\;\times \text{ 100} \end{array}$$

(4) $$\begin{array}{l} \text{Damage index}=\frac{\begin{array}{l} \sum (\text{Number of damage leaves at each level}\\ \;\times \text{Corresponding level value}) \end{array}}{\text{Total number of investigation }\times 9}\times 100 \end{array}$$

### Data Analysis

A significant difference was obtained using analysis of variance (ANOVA) by Duncan's test at a significance level of 95% with SPSS v17.0 (SPSS Inc., an IBM Company, Chicago, IL, USA), and Excel software (Microsoft Office 2019, Microsoft Corporation, Redmond, Washington, USA) was used to calculate the coefficient of variation (CV). The CV was used to show the uniformity of droplet deposition and can be presented as (Xiao et al., 2019).


(5) $$\begin{array}{l} CV=\frac{S}{\bar{X}}\times 100\%, \end{array}$$

(6) $$\begin{array}{l} S=\sqrt{\overset{n}{\underset{i=1}{\sum }}{\left(X_{i}-\bar{X}\right)}^{2}/\left(n-1\right)} \end{array}$$

where, *S* is the standard deviation (SD) of the samples in the same test group, *X*_*i*_ is the droplet density or coverage of each sampling point, $\bar{\text{X}}$ is the mean value of the droplet density or coverage in each test group, and *n* is the number of sampling points in each test group.

## Results

### Visual Photos of Droplet Deposition

**Figure 4:**
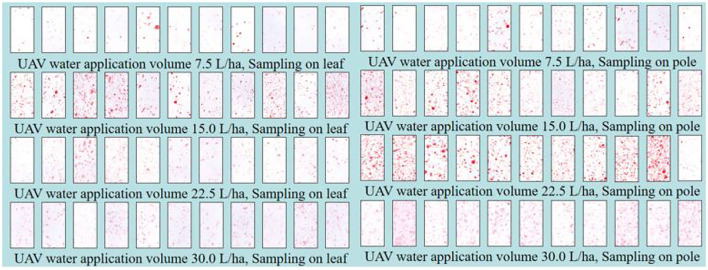
A visual aid showing deposition on the representative Kromekote® cards by UASS application.

The droplet deposition has a great effect on the control efficacy. Figure 4 is a visual photo of the droplet deposition of the MG-1P UASS with different water application volumes and sampling methods. Three qualitative conclusions can be drawn from the visual photos: (1) the water application volume has a significant effect on the droplet density and coverage; (2) the droplet density and coverage obtained by sampling on the pole were higher than those obtained by sampling on the leaf; and (3) a significant difference in the deposition was observed at different sampling points, indicating poor deposition uniformity.

### Quantitative Analysis of Deposition Characteristics

#### Effect of Sampling Methods on Droplet Deposition

**Figure 5:**
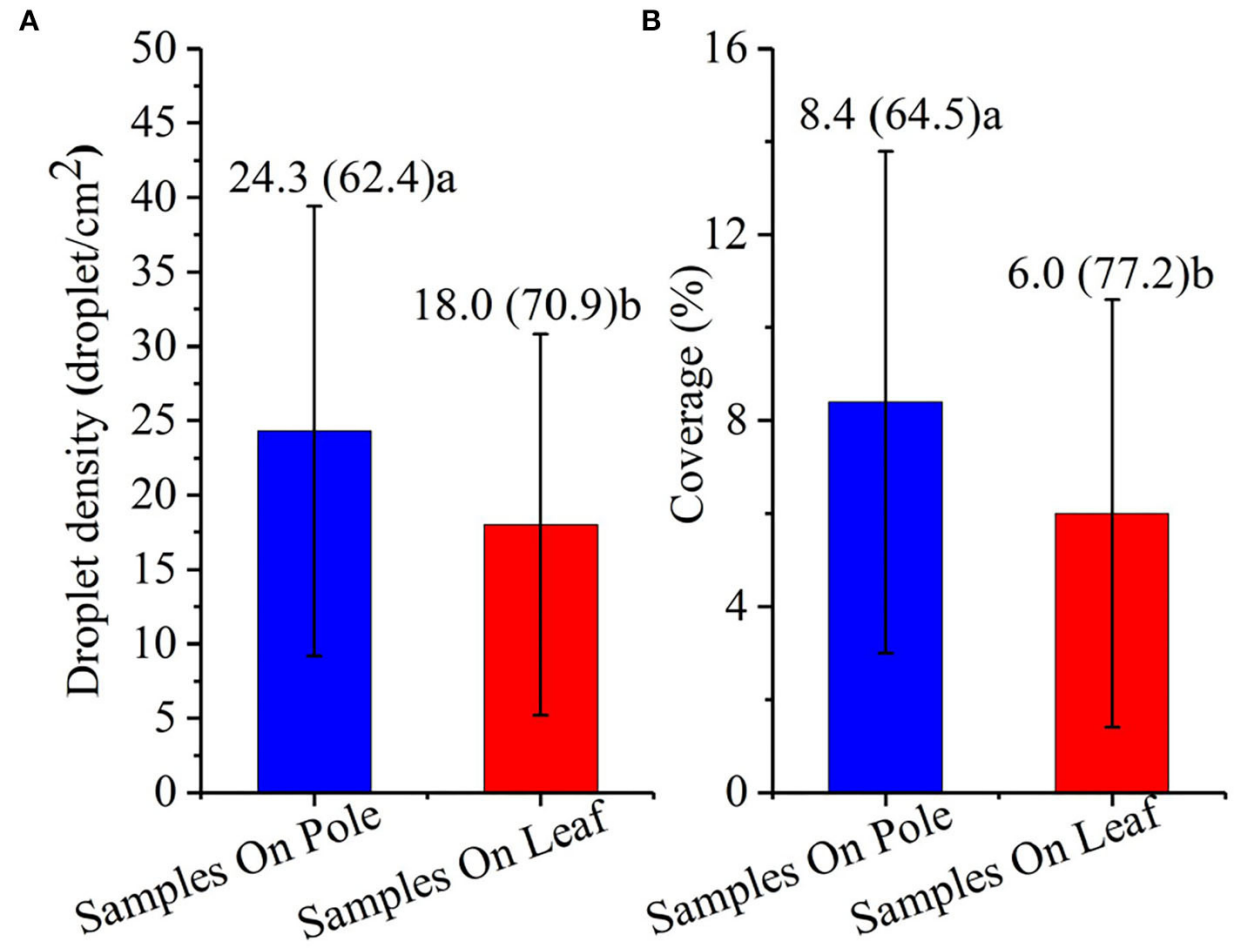
Effects of sampling pole method and sampling leaf method on deposition characteristics of droplet density and coverage. **(A)** Droplet density and **(B)** coverage. The numbers in the figure are the mean value (CV), and the different lowercase letters after the numbers indicate the significant difference, *p* < 0.01.

The droplet deposition (droplet density and coverage) values obtained by different sampling methods are shown in Figure 5. Under the water application volumes of 7.5–30.0 L/ha, the droplet density and coverage achieved by the sampling on the pole method were 24.3 droplet/cm^2^ and 8.4%, respectively; by the sampling on the leaf method, they were 18.0 droplet/cm^2^ and 6.0%, respectively. The droplet density and coverage obtained by the sampling on the pole method were 35.0 and 40.0% higher than those obtained by the sampling on the leaf method, and the difference was significant (*p* < 0.01). The CV values of deposition obtained by the two sampling methods were all higher than 60.0%, indicating that the uniformity of the deposition was poor.

#### Effect of Water Application Volumes on Droplet Deposition

**Figure 6:**
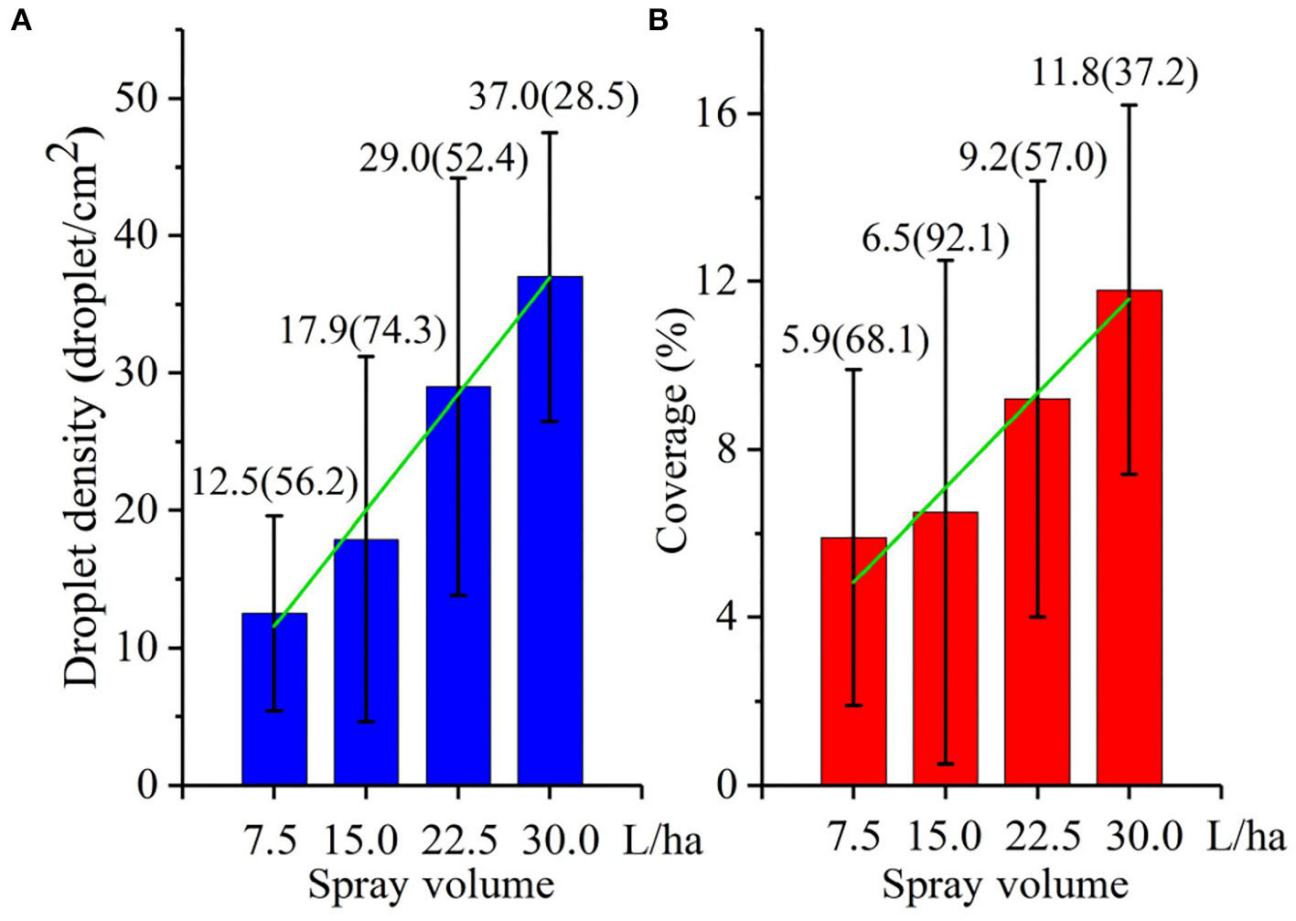
Droplet deposition at different water application volumes for various treatments. **(A)** Droplet density and **(B)** coverage. The numbers in the figure represent the mean value (CV). The green line is a fitted curve for the droplet density/coverage and water application volume.

The droplet density and coverage under different water applications are shown in Figure 6. When the water applications volume of the UASS was in the range of 7.5–30.0 L/ha, the droplet density was 12.5–37.0 droplet/cm^2^ and the coverage was 5.9–11.8%. The droplet density and coverage increased as the water application volumes increased. Through linear fitting of the data, a good linear relationship was found between the droplet deposition (droplet density and coverage) and water application volume. The coefficients of determination of the droplet density and coverage were 0.89 and 0.92, respectively.

### Control Efficacy for Fall Armyworms

#### Effect of Water Application Volumes on the Control Efficacy

**Figure 7:**
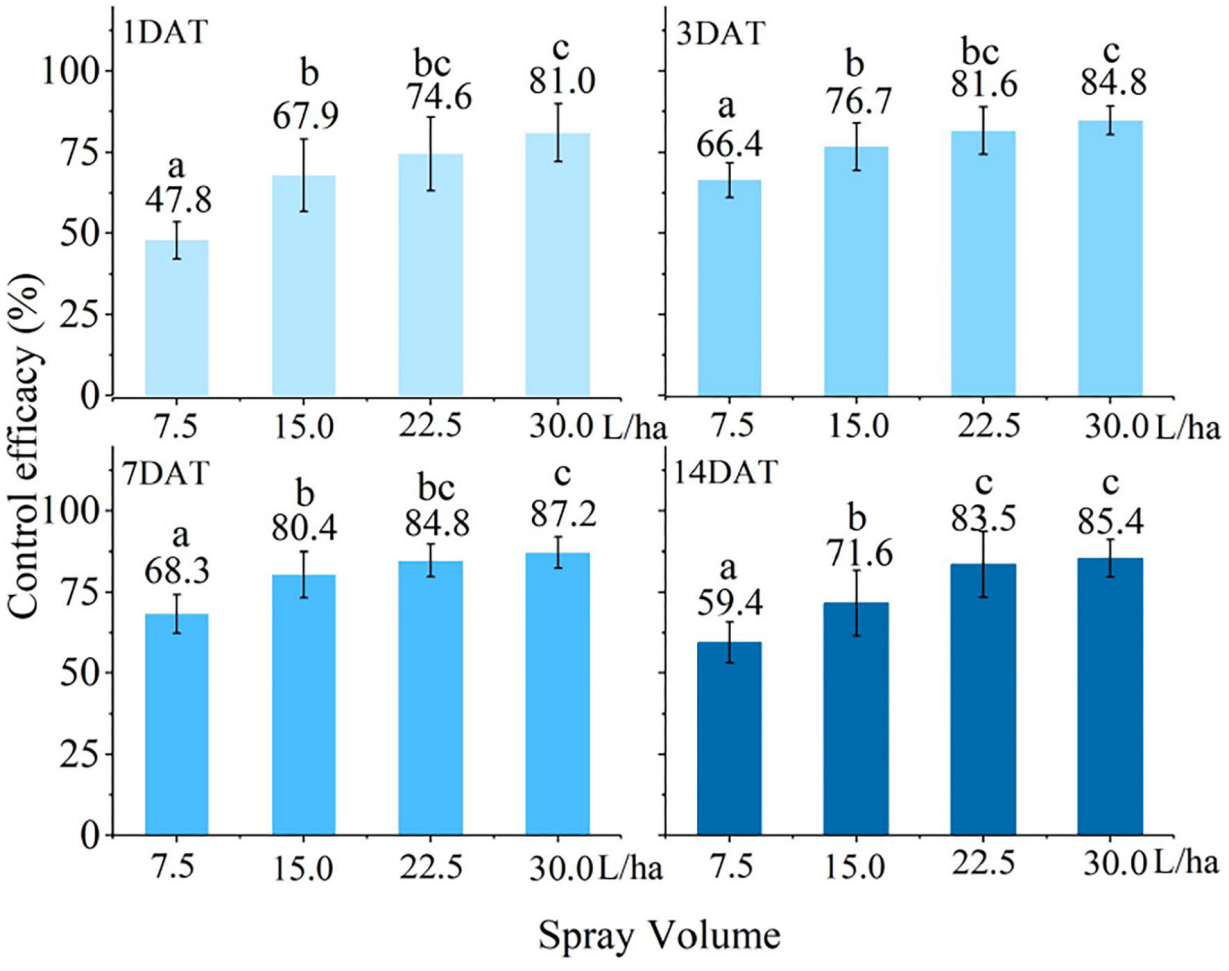
Control efficacy (%) of fall armyworm (FAW) was evaluated at 1, 3, 7, and 14 days after treatment (DAT) resulting from field-treated 25% Spinetoram water-dispersible granule (Delegate®) with an unmanned aerial spray system sprayer at four spray volumes (the different lowercase letters indicate significant results, *p* < 0.01).

The control efficacy under different water application volumes achieved by the UASS sprayer on FAW is indicated in Figure 7. From the live insect investigation results, the control efficacy significantly increased as the water application volume increased (*p* < 0.01). At 7 days after treatment (DAT), the best control efficacy was achieved at 30.0 L/ha using the UASS sprayer. However, the control efficacy of the water application volume of 30.0 and 22.5 L/ha did not differ significantly. Meanwhile, it can also be seen that the control effect of the same water application volume varies with different application days. The control efficacy gradually increased from 1 to 7 DAT and decreased from 7 to 14 DAT. The peak of the control efficacy appeared at 7 DAT. This changing trend was the same under different water application volumes.

#### Effect of Water Application Volumes on the Damage Index

**Figure 8:**
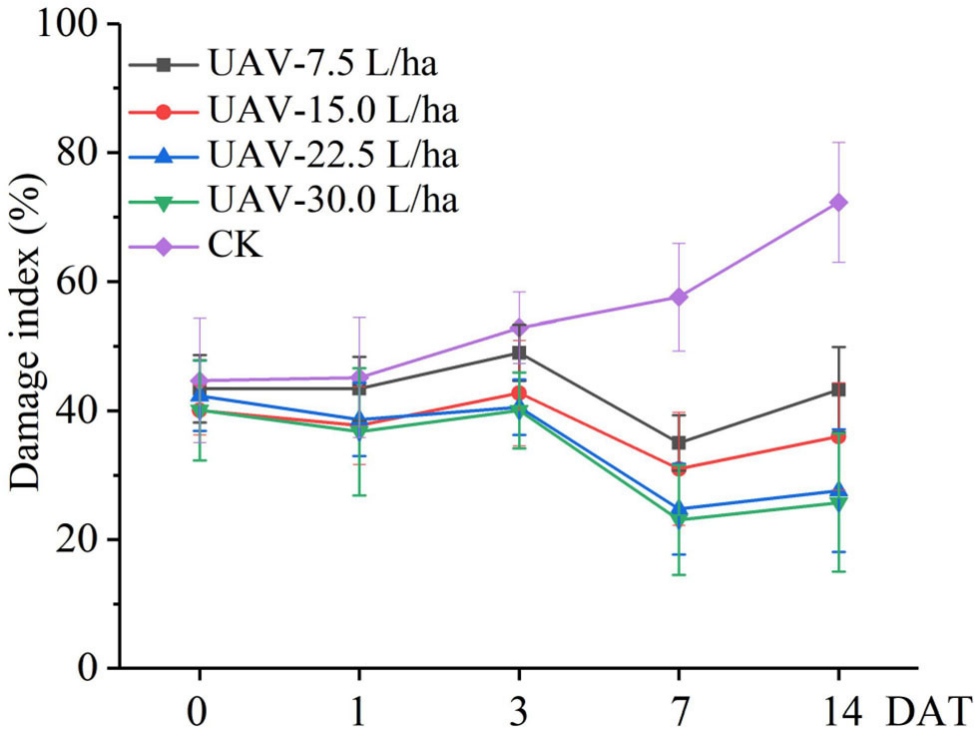
The damage index changed with the application time.

The damage index of FAW for corn when employing the UASS sprayers and a blank control at 0, 1, 3, 7, and 14 DAT is indicated in Figure 8. Before application, the damage index of each treatment area was approximately the same, which indicated that the original insect population of each treatment was similar. The trough of the damage index appeared at 7 DAT, after which the damage index began to increase, which corresponds to the results of the insect control efficacy. At 14 DAT, the blank control displayed the largest difference in the damage index compared with the treatment, which was 61.7% higher than the worst control efficacy treatment of the UASS.

## Discussion

The experimental results of this study show that there are significant differences between the two sampling methods used for droplet deposition. The results of this experiment were related to the angle of the Kromekote® card arrangement. The Kromekote® card arrangement affects the sampling efficiency of the droplet to some extent. Capri et al. (2005) measured the off-target deposition of chlorpyrifos in two fields: one field was flat and the other field was sloped. It was indicated that the flat field deposition was higher than that of the sloped field. Besides, the Kromekote® cards arranged on the sampling leaves were affected by the shielding of the leaves. Various crops have different canopy sizes, canopy shapes, foliage densities, and planting arrangements, all of which affect the droplet density and coverage (Heidary et al., 2014; Pan et al., 2016; Badules et al., 2017; Hong et al., 2017, 2018). Mostly, the upper layer of the plant canopy displays higher deposition than that of the lower layers. In a study on cotton defoliant spraying, the droplet density and coverage of the upper layer increased by 61.9 and 150.0%, respectively, compared with the lower layers (Xiao et al., 2019). The reason for this was that the upper leaves of the cotton canopy were complex and overlapping, which affected the deposition of the lower droplet. Lefrancq et al. (2013) used 51 glass Petri dishes to successfully collect the sample deposition of kresoxim-methyl in a vineyard catchment, for which the change in the deposition values was caused by the droplet being intercepted by the vine plant canopy. Of course, various sampling methods have different advantages. In this study, the sampling pole method can avoid the influence of the canopy structure and arrangement angle on droplet deposition and collect droplet deposition without bias, which will help in comparing the results obtained from different research. The sampling pole method should be used for physical characterization studies. For example, the performance of different UASS and the effects of operating parameters on droplet deposition characteristics were studied. However, it cannot replace the deposition of droplets on the leaves. The method of arranging the Kromekote® cards directly on the leaves could directly obtain the deposition of the droplets on specific leaves at different growth stages of the plant, which helped build a relationship between the droplet deposition and control efficacy. The sampling leaf method needs to include a more natural target configuration to be representative of the target structure. For example, the control effect of pests and the penetration research test of droplets.

Control efficacy experiments on corn FAW were performed with different water application volumes using UASS sprayers. The experimental results of this study show that the control efficacy gradually increased with the water application volume increased. Wang et al. (2019a) used UASS to study the control experiments of three different water application volumes (9.0, 16.8, and 28.1 L/ha) on wheat aphids. Their results were consistent with this experimental research results. However, their control efficacy results were better than this experimental result, which may be related to the operating parameters of the UASS and the droplet size. Qin et al. (2016) compared the control efficacy of low-volume spraying technology for rice planthoppers (*N. lugens*). By optimizing the spraying parameters of the UASS, the control efficacy was improved. Chen et al. (2020) used three nozzles with different droplet sizes to study the effects of different droplet sizes on the rice planthopper control efficacy. The results show that the selection of nozzles with smaller atomizing particle sizes for UASS can improve the control efficacy of rice planthoppers (*N. lugens*). In addition, different results were found by other researchers. Roehrig et al. (2018) tested spray volumes between 40 and 160 L/ha and verified that the 130 L/ha was higher than the others for soybean yield, being statistically similar to the 160 L/ha. Sánchez-Hermosilla et al. (2011) reduced application volumes from 1,000 to 500 L/ha and improved crop control product application in tomatoes by altering the spray gun (900 and 1,800 L/ha) on the vertical spray boom. Berger-Neto et al. (2017) compared two spray volumes of 100 and 200 L/ha, and concluded that spray volume did not affect the control of white mold in soybean. Garcerá et al. (2014) also found that spray application volumes (11.74, 17.65, and 32.21 L/ha) did not affect two of the organophosphate insecticides controlling California red scale infestation. Their results were not consistent with our research results. This may be caused by excessive spray volume. Wang et al. (2019a) conducted experiments on wheat with different sprayers, and the results proved that high-volume (225 and 450 L/ha) spraying easily leads to run-off and lower deposition, thereby reducing the control efficacy of high-volume. Thus, it is not necessary to use high-volume spraying, as a certain number of spray volumes achieve good efficacy.

In this study, the number of live insects decreased significantly by 1 DAT, but the damage index did not change much. This may be because the investigation method of the damage index has hysteresis compared with the investigation method of the insect control efficacy. The control effect was best on the 7DAT. At 14 DAT, the control efficacy was reduced, indicating that the insecticides had a shelf life of fewer than 14 days, increasing the number of live insects. The control efficacy of the water application volume of 30.0 and 22.5 L/ha did not differ significantly. Considering work efficiency, a water application volume of 22.5 L/ha is recommended for field operation. The control efficacy (84.8%) of the UASS sprayer meets basic field control requirements, but the UASS has the advantage of a high efficiency in comparison with the large-capacity spray, which has an important role in the rapid control of explosive pests. Of course, further work will be to continuously improve the control efficacy of the UASS by adding spraying adjuvants or optimizing the spraying system.

## Conclusions

In this study, four different water application volumes were used for pesticide application in the cornfield. The droplet deposition characteristics of different sampling methods and the control efficacy for corn FAW using different water application volumes were compared in this research. The conclusions are as follows:

1. The droplet density and coverage were affected by the sampling method;
2. There was a good linear relationship between the droplet deposition (droplet density or coverage) and water application volumes;
3. The control efficacy increased and the damage index decreased with the increase of water application volumes. When using plant protection UASS in the field, it is recommended to use 22.5 L/ha of water application volume.

The experiments demonstrated the feasibility of UASS sprayers in controlling corn FAW fields. However, the control efficacy of UASS needs to be further improved. Due to the poor deposition uniformity, effective measures, such as adding an adjuvant in the tank or optimizing the spraying system, which can improve the deposition uniformity, will be needed in the future.

## Data Availability Statement

The original contributions presented in the study are included in the article/Supplementary Material, further inquiries can be directed to the corresponding authors.

## Author Contributions

CSh designed the experiments and wrote the manuscript. JWu, SC, CSo, and JWa carried out the experiments. CSo and HW collected material data and analyzed experimental results. GW and YL supervised and revised the manuscript. All authors contributed to the article and approved the submitted version.

## Funding

This study was supported by Top Talents Program for One Case One Discussion of Shandong Province, Academy of Ecological Unmanned Farm (Grant No. 2019ZBXC200), Shandong Province Natural Science Foundation (Grant No. ZR2021QC154), and Young Innovative Talents Project of Regular Institutions of Higher Education of Guangdong Province (Grant No. 2018KQNCX020).

## Conflict of Interest

JWu and HW were employed by Corteva Agroscience Technology (Shanghai) Co., Ltd. The remaining authors declare that the research was conducted in the absence of any commercial or financial relationships that could be construed as a potential conflict of interest.

## Publisher's Note

All claims expressed in this article are solely those of the authors and do not necessarily represent those of their affiliated organizations, or those of the publisher, the editors and the reviewers. Any product that may be evaluated in this article, or claim that may be made by its manufacturer, is not guaranteed or endorsed by the publisher.

## References

[B1] American Society of Agricultural Engineers (1995). Calibration and Distribution Pattern Testing of Agricultural Aerial Application Equipment. State of Michigan: American Society of Agricultural Engineers. p. 229–232.

[B2] Badules J. Vidal M. Bon,é A. Llop J. Salcedo R. Gil E.. (2017). Comparative study of CFD models of the air flow produced by an air-assisted sprayer adapted to the crop geometry. Comp. Electron. Agric. 149, 166–174. 10.1016/j.compag.2017.09.026

[B3] Berger-Neto A. Jaccoud-Filho D. D. S. Wutzki C. R. Tullio H. E. Pierre M. L. C. Manfron F.. (2017). Effect of spray droplet size, spray volume and fungicide on the control of white mold in soybeans. Crop Prot. 92, 190–197. 10.1016/j.cropro.2016.10.016

[B4] Berner B. Chojnacki J. (2017). “Use of drones in crop protection. “Farm Machinery and Processes Management in Sustainable Agriculture”,” in *Proceedings of IX International Scientific Symposium, Lublin, Poland*.

[B5] Brain R. A. Perine J. Cooke C. Ellis C. B. Harrington P. Lane A.. (2017). Evaluating the effects of herbicide drift on nontarget terrestrial plants: a case study with mesotrione. Environ. Toxicol. Chem. 36, 2465–2475. 10.1002/etc.3786 28262983

[B6] Capri E. Balderacchi M. Yon D. Reeves G. H. (2005). Deposition and dissipation of Chlorpyrifos in surface water following vineyard applications in Northern Italy. Environ. Toxicol. Chem. 24, 852–860. 10.1897/04-151R.1 15839559

[B7] Casmuz A. Juárez M. L. Socías M. G. Prieto S. Medina S. Willink E.. (2010). Revisión de los hospederos del gusano cogollero del maíz, *Spodoptera frugiperda* (Lepidoptera: Noctuidae). *Revista de la Sociedad Entomológica Argentina*. 69, 209–231. Available online at: https://www.redalyc.org/articulo.oa?id=322028487010

[B8] Chen P. C. Lan Y. B. Huang X. Y. Qi H. X. Wang G. B. Wang J.. (2020). Droplet deposition and control of planthoppers of different nozzles in two-stage rice with a quadrotor unmanned aerial vehicle. Agronomy 10, 303. 10.3390/agronomy10020303

[B9] Cui L. Rui C. H. Li Y. P. Wang Q. G. Yang D. B. Yan X. J.. (2019). Research and application of chemical control technology against *Spodoptera frugiperda* (Lepidoptera:Noctuidae) in foreign countries. Plant Prot. 45, 7–13.

[B10] Davis F. M. Ng S. S. Williams W. P. (1992). Visual rating scales for screening whorl-stage corn for resistance to fall armyworm. Tech. Bull. Mississippi Agric. Forest. Exp. Station. 186, 1–9.

[B11] Ebert T. A. Taylor R. A. Downer R. A. Hall F. R. (1999). Deposit structure and efficacy of pesticide application. 1: Interactions between deposit size, toxicant concentration and deposit number. Pest. Sci. 55, 783–792. 10.1002/(SICI)1096-9063(199908)55:8<783::AID-PS973>3.0.CO;2-D

[B12] Faiçal B. S. Costa F. G. Pessin G. Ueyama J. Freitas H. Colombo A.. (2014). The use of unmanned aerial vehicles and wireless sensor networks for spraying pesticides. J. Syst. Arch. 60, 393–404. 10.1016/j.sysarc.2014.01.004

[B13] Faiçal B. S. Freitas H. Gomes P. H. Mano L. Y. Pessin G. Carvalho A.. (2017). An adaptive approach for UAV-based pesticide spraying in dynamic environments. Comp. Electron. Agric. 138, 210–223. 10.1016/j.compag.2017.04.011

[B14] Ferreira Filho J. B. Alves L. R. A. Gottardo L. C. B. Georgino M. (2010). “Dimensionamento do custo economico representado por Spodoptera frugiperda na cultura do milho no Brasil,” in 48 Congresso Sociedade Brasileira de Economia. Administracao e Sociologia Rural.

[B15] Garcerá C. Moltó E. Chueca P. (2014). Factors influencing the efficacy of two organophosphate insecticides in controlling California red scale, *Aonidiella aurantii* (Maskell). A basis for reducing spray application volume in Mediterranean conditions. Pest Manage. Sci. 70, 28–38. 10.1002/ps.3515 23404841

[B16] Goergen G. Kumar P. L. Sankung S. B. Togola A. Tamò M. (2016). First report of outbreaks of the fall armyworm *Spodoptera frugiperda* (J E Smith) (Lepidoptera, Noctuidae), a new alien invasive pest in west and central Africa. PLoS ONE 11, e0165632. 10.1371/journal.pone.0165632 27788251 PMC5082806

[B17] Guo H. Zhou J. Liu F. He Y. Huang H. Wang H. Y. (2020). Application of machine learning method to quantitatively evaluate the droplet size and deposition distribution of the UAV spray nozzle. Appl. Sci. 10, 1759. 10.3390/app10051759

[B18] Harrison R. D. Thierfelder C. Baudron F. Chinwada P. Midega C. Schaffner U.. (2019). Agro-ecological options for fall armyworm (*Spodoptera frugiperda JE Smith*) management: providing low-cost, small holder friendly solutions to an invasive pest. J. Environ. Manage. 243, 318–330. 10.1016/j.jenvman.2019.05.011 31102899

[B19] He L. Wang G. B. Hu T. Meng Y. H. Yan X. J. Yuan H. Z. (2017). Influences of spray adjuvants and spray volume on the droplet deposition distribution with unmanned aerial vehicle (UAV) spraying on rice. J. Plant Prot. 44, 1046–1052. (In Chinese). 10.13802/j.cnki.zwbhxb.2017.2016147

[B20] Heidary M. A. Douzals J. P. Sinfort C. Vallet A. (2014). Influence of spray characteristics on potential spray drift of field crop sprayers: a literature review. Crop Prot. 63, 120–130. 10.1016/j.cropro.2014.05.006

[B21] Hong S. W. Zhao L. Zhu H. (2017). CFD simulation of airflow inside tree canopies discharged from air-assisted sprayers. Comp. Electron. Agric. 149, 121–132. 10.1016/j.compag.2017.07.011

[B22] Hong S. W. Zhao L. Y. Zhu H. P. (2018). SAAS, a computer program for estimating pesticide spray efficiency and drift of air-assisted pesticide applications. Comp. Electron. Agric. 155, 58–68. 10.1016/j.compag.2018.09.031

[B23] Hruska A. Gould F. (1997). Fall armyworm (Lepidoptera: Noctuidae) and *Diatraea lineolata* (Lepidoptera:Pyralidae): impact of larval population level and temporal occurrence on maize yield in nicaragua. J. Econ. Entomol. 90, 611–622. 10.1093/jee/90.2.611

[B24] Huang Y. B. Thomson S. J. Hoffmann W. C. Lan Y. B. Fritz B. K. (2013). Development and prospect of unmanned aerial vehicle technologies for agricultural production management. Int. J. Agric. Biol. Eng. 6, 1–10. 10.3965/j.ijabe.20130603.001

[B25] Jeon H. Y. Tian L. (2010). Machine vision instrument to measure spray droplet sizes. J. Biosyst. Eng. 35, 443–449. 10.5307/JBE.2010.35.6.443

[B26] Jing D. Guo J. Jiang Y. Zhao J. Sethi A. He K.. (2019). Initial detections and spread of invasive *Spodoptera frugiperda* in China and comparisons with other noctuid larvae in cornfield using molecular techniques. Insect Sci. 27, 780–790. 10.1111/1744-7917.12700 31209955 PMC7317731

[B27] Johnson J. S.. (1987). Migration and the life history strategy of the fall armyworm, *Spodoptera frugiperda* in the western hemisphere. Int. J. Trop. Insect Sci. 8, 543–549. 10.1017/S1742758400022591

[B28] Kharim M. N. A. Wayayok A. Shariff A. R. M. Abdullah A. F. Husin E. M. (2019). Droplet deposition density of organic liquid fertilizer at low altitude UAV aerial spraying in rice cultivation. Comp. Electron. Agric. 167, 105045. 10.1016/j.compag.2019.105045

[B29] Lan Y. B. Chen S. D. (2018). Current status and trends of plant protection UAV and its spraying technology in China. Int. J. Precis. Agric. Aviat. 1, 1–9. 10.33440/j.ijpaa.20180101.0002

[B30] Lefrancq M. Imfeld G. Payraudeau S. Millet M. (2013). Kresoxim methyl deposition, drift and runoff in a vineyard catchment. Sci. Total Environ. 442, 503–508. 10.1016/j.scitotenv.2012.09.082 23201604

[B31] Lu H. Li F. Zhu X. M. Tang J. H. Lyu Q. B. Wu S. Y. (2021). Preliminary study on the effect of using unmanned aerial vehicles (UAVs) to control *Spodoptera frugiperda*. Entomol. Res. 51, 453–461. 10.1111/1748-5967.12538

[B32] Meng Y. H. Han Y. X. Liang Z. Su J. Lan Y. B. (2019). Harvest-aid application strategy in different cotton planting densities using UAV: effects of dosage and application frequency on defoliation efficacy, boll opening rate, fiber quality, and lint cotton yield. Int. J. Precis. Agric. Aviat. 2, 31–41. 10.33440/j.ijpaa.20190201.0027

[B33] Mitchell E. R.. (1979). Fall armyworm symposium: preface. Fla. Entomol. 62, 81. 10.2307/3494085

[B34] Montezano D. Specht A. Sosa-G'omez D. Roque-Specht V. Sousa-Silva J. PaulaMoraes S.. (2018). Host plants of spodoptera frugiperda (Lepidoptera: Noctuidae) in the Americas. Afr. Entomol. 26, 286–300. 10.4001/003.026.0286

[B35] Pachuta A. Boguslawa B. Chojnacki J. (2018). “Evaluation of liquid transverse distribution under a twin spray jet installed on a drone,” in MendelNet Conference Bron 2018, Brno, Czech Republic.

[B36] Pan Z. Lie D. Qiang L. He S. L. Yi S. Liu Y. D.. (2016). Effects of citrus tree-shape and spraying height of small unmanned aerial vehicle on droplet distribution. Int. J. Agric. Biol. Eng. 9, 45–52. 10.3965/j.ijabe.20160904.2178

[B37] Phani K. K. Mohan V. K. Kamakshi N. Dakshina M. K. Mohan R. K. (2021). Field efficacy of selected insecticides against invasive pest, fall armyworm *Spodoptera frugiperda* (j. E. Smith) on maize crop. Pharma Innovat. J. 10, 884–889. 10.1653/024.103.0211

[B38] Qin W. C. Qiu B. J. Xue X. Y. Chen C. C. Xu Z. F. Zhou Q. Q. (2016). Droplet deposition and control effect of insecticides sprayed with an unmanned aerial vehicle against plant hoppers. Crop Prot. 85, 79–88. 10.1016/j.cropro.2016.03.018

[B39] Qin W. C. Xue X. Y. Zhou Q. Q. Cai C. Wang G. Jin Y. (2018). Use of RhB and BSF as fluorescent tracers for determining pesticide spray distribution. Anal. Methods UK 10, 4073–4078. 10.1039/C8AY01198B

[B40] Roehrig R. Boller W. Forcelini C. A. Chechi A. (2018). Use of surfactant with different volumes of fungicide applications in soybean culture. Engenharia Agrícola 38, 577–589. 10.1590/1809-4430-eng.agric.v38n4p577-589/20182018

[B41] Sánchez-Hermosilla J. Rincón V. J. Páez F. Agüera F. Carvajal F. (2011). Field evaluation of a self-propelled sprayer and effects of the application rate on spray deposition and losses to the ground in greenhouse tomato crops. Pest Manag. Sci. 67, 942–947. 10.1002/ps.2135 21394883

[B42] Wang G. B. Lan Y. B. Qi H. X. Chen P. C. Hewitt A. Han Y. X. (2019a). Field evaluation of an unmanned aerial vehicle (UAV) sprayer: effect of spray volume on deposition and the control of pests and disease in wheat. Pest Manag. Sci. 75, 1546–1555. 10.1002/ps.5321 30620130

[B43] Wang G. B. Lan Y. B. Yuan H. Z. Qi H. X. Chen P. C. Ouyang F.. (2019b). Comparison of spray deposition, control efficacy on wheat aphids and working efficiency in the wheat field of the unmanned aerial vehicle with boom sprayer and two conventional knapsack sprayers. Appl. Sci. 9, 218. 10.3390/app9020218

[B44] Westbrook J. K. Nagoshi R. N. Meagher R. L. Fleischer S. J. Jairam S. (2015). Modeling seasonal migration of fall armyworm moths. Int. J. Biometeorol. 60, 255–267. 10.1007/s00484-015-1022-x 26045330

[B45] Xiao Q. G. Xin F. Lou Z. X. Zhou T. T. Wang G. B. Han X. Q.. (2019). Effect of aviation spray adjuvants on defoliant droplet deposition and cotton defoliation efficacy sprayed by unmanned aerial vehicles. Agronomy 9, 217. 10.3390/agronomy9050217

[B46] Xin F. Zhao J. Zhou Y. T. Wang G. B. Han X. Q. Fu W.. (2018). Effects of dosage and spraying volume on cotton defoliants efficacy: a case study based on application of unmanned aerial vehicles. Agronomy 8, 85. 10.3390/agronomy8060085

[B47] Yan X. J. Yuan H. Z. Chen Y. X. Shi X. Liu X. H. Wang Z. Y.. (2021). Broadcasting of tiny granules by drone to mimic liquid spraying for the control of fall armyworm (*Spodoptera frugiperda*). *Pest Manag. Sci*. 78, 43–51. 10.1002/ps.6604 34405509

[B48] Zhang D. Y. Chen L. P. Zhang R. R. Hoffmann W. C. Xu G. Lan Y. B.. (2015). Evaluating effective swath width and droplet distribution of aerial spraying systems on M-18B and thrush 510G airplanes. Int. J. Agric. Biol. Eng. 8, 21–30. 10.3965/j.ijabe.20150802.1493

[B49] Zhang P. Wang K. J. Lyu Q. He S. L. Yi S. L. Xie R. J.. (2017). Droplet distribution and control against citrus leafminer with UAV spraying. Int. Conf. Robot. Autom. 32, 299–307. 10.2316/Journal.206.2017.3.206-4980

