# Supplementary material for: Control Efficacy and Deposition Characteristics of an Unmanned Aerial Spray System Low-Volume Application on Corn Fall Armyworm Spodoptera frugiperda
Source: Front Plant Sci. 2022 Sep 13;13:900939. doi: 10.3389/fpls.2022.900939 (PMC9514045; doi:10.3389/fpls.2022.900939)
Supplement: Supplementary file 1 [file Data_Sheet_1.doc]

Introduction：Table 1 and Table 2 are the deposition data collected at 11 sampling points under four different water consumptions on the sampling pole and sampling leaf. Table 3 and Table 4 are the data of the control efficacy and damage index of different treatments. The the experiment was repeated 3 times for each treatment.

Table 1 Different treatments deposition on the sampling pole

| Repeat | Water application volume | 7.5 L/ha | | 15.0 L/ha | | 22.5 L/ha | | 30.0 L/ha | |
| --- | --- | --- | --- | --- | --- | --- | --- | --- | --- |
|  | Sampling point | Droplet density (droplet/cm^2^) | Coverage  (%) | Droplet density (droplet/cm^2^) | Coverage  (%) | Droplet density (droplet/cm^2^) | Coverage  (%) | Droplet density (droplet/cm^2^) | Coverage  (%) |
| Repeat 1 | 1 | 7.4 | 1.2 | 4.7 | 1.1 | 34.6 | 10.93 | 11.5 | 2.3 |
|  | 2 | 5.2 | 9.7 | 24.7 | 12.2 | 35.9 | 10.23 | 55.9 | 20.1 |
|  | 3 | 20.6 | 4 | 28.5 | 14.58 | 59.4 | 18.8 | 43.2 | 13.2 |
|  | 4 | 16.6 | 4.1 | 34.7 | 18.8 | 14.8 | 5.01 | 35.8 | 15.4 |
|  | 5 | 11.3 | 8.4 | 12.4 | 3.1 | 22.4 | 7.65 | 38.9 | 12.1 |
|  | 6 | 1.2 | 0.2 | 16.2 | 6.1 | 23.3 | 4.59 | 37.8 | 11.2 |
|  | 7 | 21 | 7.8 | 10.6 | 4.5 | 32.9 | 5.81 | 26.6 | 7.8 |
|  | 8 | 0.8 | 10.1 | 14.6 | 4.4 | 19.5 | 6.3 | 28.5 | 10.1 |
|  | 9 | 23.7 | 12.1 | 15.9 | 3.2 | 30.6 | 10.79 | 38.8 | 12.1 |
|  | 10 | 0.6 | 1.8 | 32.4 | 10.1 | 51.3 | 16.4 | 43.8 | 15.4 |
|  | 11 | 2.8 | 1.7 | 2.3 | 0.5 | 5.8 | 0.87 | 48.8 | 16.4 |
| Repeat 2 | 1 | 16.3 | 0.1 | 2.1 | 0.5 | 50.4 | 20.1 | 55.8 | 20.2 |
|  | 2 | 16.6 | 0.11 | 1.9 | 0.55 | 30.7 | 12.45 | 51.9 | 15.8 |
|  | 3 | 14.9 | 9.6 | 35 | 12.1 | 29.4 | 11.81 | 41.2 | 13.4 |
|  | 4 | 18.5 | 12.8 | 45.3 | 16.7 | 17.6 | 3.62 | 27.4 | 7.6 |
|  | 5 | 21.7 | 9.8 | 2.2 | 0.1 | 39.2 | 15.22 | 31.8 | 6.8 |
|  | 6 | 8.6 | 4.2 | 12.1 | 0.32 | 38.8 | 12.35 | 33.5 | 8.3 |
|  | 7 | 15.8 | 8.6 | 33.8 | 12.26 | 40.4 | 13.1 | 28.6 | 7.1 |
|  | 8 | 11.7 | 5.2 | 2.1 | 1.05 | 2.2 | 0.96 | 33.5 | 9.3 |
|  | 9 | 12.4 | 4.3 | 20.9 | 6.92 | 16.1 | 5.14 | 28.4 | 9.8 |
|  | 10 | 14.5 | 6.4 | 7.8 | 2.59 | 5.8 | 2.27 | 32.5 | 13.5 |
|  | 11 | 14.1 | 6.6 | 5.6 | 2.2 | 38 | 15.17 | 41.5 | 12.6 |
| Repeat 3 | 1 | 20.1 | 8.4 | 25.2 | 12.7 | 54.2 | 17.1 | 42.8 | 14.1 |
|  | 2 | 23.2 | 9.6 | 34.8 | 14.3 | 34.1 | 12.3 | 16.4 | 4.8 |
|  | 3 | 4.2 | 0.8 | 29.6 | 9.2 | 38.8 | 12.2 | 35.2 | 11.1 |
|  | 4 | 15.5 | 7.1 | 4.2 | 0.8 | 8.3 | 4.8 | 26.8 | 7.8 |
|  | 5 | 12.2 | 3.3 | 30.2 | 6.7 | 3.4 | 1.8 | 32.3 | 10.1 |
|  | 6 | 20.6 | 14 | 3.2 | 0.1 | 33.8 | 8.6 | 56.6 | 21.8 |
|  | 7 | 0.8 | 0.1 | 15.2 | 2.2 | 44.3 | 11.1 | 32.6 | 12.2 |
|  | 8 | 3.7 | 1.3 | 36.1 | 17.4 | 13.1 | 4.2 | 46.5 | 14.1 |
|  | 9 | 10.4 | 7.2 | 8.8 | 4.4 | 18.2 | 5.2 | 48.2 | 16.1 |
|  | 10 | 13.8 | 6.4 | 34.6 | 14.2 | 34.7 | 8.4 | 33.8 | 9.8 |
|  | 11 | 13.1 | 6.5 | 4.2 | 0.1 | 36.6 | 8.1 | 35.2 | 8.2 |

Table 2 Different treatments deposition on the sampling leaf

| Repeat | Water application volume | 7.5 L/ha | | 15.0 L/ha | | 22.5 L/ha | | 30.0 L/ha | |
| --- | --- | --- | --- | --- | --- | --- | --- | --- | --- |
|  | Sampling point | Droplet density (droplet/cm^2^) | Coverage (%) | Droplet density (droplet/cm^2^) | Coverage (%) | Droplet density (droplet/cm^2^) | Coverage (%) | Droplet density (droplet/cm^2^) | Coverage (%) |
| Repeat 1 | 1 | 1.7 | 0.25 | 5.2 | 1.2 | 3.3 | 1.15 | 22 | 3.2 |
|  | 2 | 4.9 | 0.6 | 16.2 | 6.2 | 19.9 | 4.78 | 2.6 | 0.13 |
|  | 3 | 22.1 | 9.2 | 28.5 | 10.6 | 38.9 | 15.83 | 4.4 | 1.4 |
|  | 4 | 8.9 | 4.4 | 21.8 | 6.9 | 33.2 | 11.26 | 32.5 | 13.2 |
|  | 5 | 14.1 | 5.1 | 26.8 | 11.8 | 2.5 | 0.25 | 25.8 | 10.6 |
|  | 6 | 5.2 | 1.41 | 4.5 | 2.2 | 30.6 | 6.11 | 26.2 | 8.4 |
|  | 7 | 1.7 | 0.7 | 16.1 | 5.2 | 28.3 | 4.4 | 9.5 | 7.3 |
|  | 8 | 20.4 | 9.2 | 12.2 | 4.2 | 24 | 3.97 | 22 | 9.6 |
|  | 9 | 2.8 | 0.47 | 22.5 | 6.7 | 9.3 | 2.17 | 25.8 | 5.1 |
|  | 10 | 5.1 | 2.11 | 2.4 | 0.4 | 33.9 | 5.36 | 33.9 | 11.2 |
|  | 11 | 13.4 | 3.1 | 36.7 | 13.1 | 13.7 | 3.42 | 29 | 11.1 |
| Repeat 2 | 1 | 11.3 | 5.4 | 3.7 | 0.9 | 6.9 | 2.13 | 51.8 | 15.4 |
|  | 2 | 8.9 | 1.8 | 4.1 | 1.2 | 22.8 | 9.7 | 2.7 | 0.4 |
|  | 3 | 22.6 | 13.1 | 42.8 | 12.3 | 34.5 | 14.87 | 37.9 | 13.6 |
|  | 4 | 8.6 | 1.8 | 44.4 | 13.4 | 15.7 | 4.9 | 8.4 | 1.3 |
|  | 5 | 5.3 | 1.4 | 1.7 | 0.6 | 10.7 | 4.52 | 23.3 | 6.3 |
|  | 6 | 12.2 | 9.8 | 0.6 | 0.1 | 39 | 13.29 | 2.5 | 1 |
|  | 7 | 6.7 | 1.2 | 4.1 | 2.5 | 39.6 | 16.36 | 44.2 | 9.2 |
|  | 8 | 7.8 | 1.3 | 7 | 0.5 | 33.3 | 12.6 | 29.5 | 9.3 |
|  | 9 | 9.2 | 2.4 | 8 | 3.4 | 12.6 | 6.95 | 22.9 | 4.8 |
|  | 10 | 14.8 | 10.8 | 29.8 | 12.4 | 8.7 | 3.09 | 35.7 | 13.2 |
|  | 11 | 9.7 | 2.9 | 10.8 | 4.2 | 20.8 | 6.8 | 41.7 | 12.8 |
| Repeat 3 | 1 | 12.7 | 7.2 | 35.2 | 11.3 | 20.4 | 2.7 | 38.2 | 16.2 |
|  | 2 | 3.8 | 1.2 | 25.4 | 9.4 | 13.6 | 4.2 | 36.4 | 12.1 |
|  | 3 | 5.2 | 0.9 | 4.6 | 0.8 | 6.7 | 1.1 | 22.4 | 5.3 |
|  | 4 | 2.1 | 0.2 | 8.2 | 2.2 | 1.4 | 0.2 | 11.8 | 4.5 |
|  | 5 | 15.2 | 4.1 | 1.2 | 0.2 | 36.2 | 11.8 | 26.2 | 9.4 |
|  | 6 | 3.9 | 1.1 | 0.8 | 0.1 | 19.5 | 9.7 | 2.6 | 0.8 |
|  | 7 | 4.5 | 1.4 | 14.5 | 4.2 | 39.8 | 12.4 | 32.5 | 14.3 |
|  | 8 | 12.6 | 6.6 | 20.5 | 6.2 | 26.5 | 8.6 | 24.8 | 8.4 |
|  | 9 | 20.5 | 7.6 | 24.4 | 7.8 | 28.1 | 7.3 | 10.9 | 3.2 |
|  | 10 | 18.2 | 11.2 | 36.1 | 11.2 | 29.4 | 8.4 | 24.8 | 8.2 |
|  | 11 | 7.2 | 1.1 | 8.3 | 2.6 | 12.8 | 4.6 | 36.4 | 11.2 |

Table 3 Control efficacy at different water application volumes for various treatments.

| Water application volume | | 7.5L/ha | 15.0 L/ha | 22.5L/ha | 30.0L/ha | 7.5L/ha | 15.0 L/ha | 22.5L/ha | 30.0L/ha |
| --- | --- | --- | --- | --- | --- | --- | --- | --- | --- |
| Application time | | 1 day after application | | | | 7 day after application | | | |
| Control efficacy (%) | Repeat 1 | 46.4 | 78.3 | 89.1 | 81.2 | 76.1 | 70.9 | 89.4 | 82.6 |
|  |  | 40.4 | 81.4 | 79.5 | 80.8 | 67.2 | 81.9 | 85.5 | 83.5 |
|  | Repeat 2 | 41.9 | 64.8 | 79.8 | 87.6 | 59.0 | 72.8 | 82.0 | 86.4 |
|  |  | 54.5 | 50.0 | 62.2 | 85.7 | 69.6 | 85.7 | 76.4 | 91.2 |
|  | Repeat 3 | 52.5 | 68.3 | 59.5 | 64.1 | 72.8 | 88.9 | 86.2 | 84.7 |
|  |  | 50.7 | 64.5 | 77.5 | 86.7 | 65.1 | 82.4 | 89.1 | 94.6 |
| Application time |  | 3 day after application | | | | 14 day after application | | | |
| Control efficacy (%) | Repeat 1 | 57.4 | 77.4 | 86.6 | 85.4 | 48.1 | 70.3 | 96.6 | 87.6 |
|  |  | 62.9 | 62.6 | 83.8 | 83.6 | 61.0 | 62.3 | 91.9 | 77.8 |
|  | Repeat 2 | 68.9 | 81.3 | 82.0 | 82.9 | 65.5 | 65.2 | 87.5 | 81.2 |
|  |  | 69.4 | 75.4 | 67.5 | 91.9 | 61.2 | 63.4 | 71.4 | 89.6 |
|  | Repeat 3 | 72.0 | 81.6 | 87.4 | 79.0 | 56.6 | 82.9 | 73.5 | 83.3 |
|  |  | 68.1 | 81.9 | 82.5 | 86.1 | 64.1 | 85.4 | 80.2 | 93.1 |

Table 4 Damage index at different water application volumes for various treatments.

| Water application volume | | 7.5L/ha | 15.0 L/ha | 22.5L/ha | 30.0L/ha | CK | 7.5L/ha | 15.0 L/ha | 22.5L/ha | 30.0L/ha | CK |
| --- | --- | --- | --- | --- | --- | --- | --- | --- | --- | --- | --- |
| Application time | | 0 day after application | | | | | 7 day after application | | | | |
| Damage index (%) | Repeat 1 | 43.4 | 39.4 | 33.3 | 48.1 | 38.9 | 36.4 | 45.5 | 16.2 | 34.3 | 63.0 |
|  |  | 39.4 | 46.5 | 47.6 | 50.6 | 55.6 | 39.4 | 25.3 | 22.2 | 30.3 | 55.6 |
|  | Repeat 2 | 35.4 | 35.4 | 39.7 | 32.1 | 36.1 | 34.3 | 38.4 | 34.3 | 21.2 | 42.2 |
|  |  | 46.5 | 40.4 | 41.3 | 40.7 | 51.9 | 39.4 | 26.3 | 32.3 | 17.2 | 44.4 |
|  | Repeat 3 | 46.5 | 37.4 | 47.6 | 34.6 | 55.6 | 32.3 | 25.3 | 22.2 | 24.2 | 46.7 |
|  |  | 49.5 | 41.4 | 44.4 | 34.4 | 36.1 | 28.3 | 25.3 | 21.2 | 11.1 | 57.8 |
| Application time | | 1 day after application | | | | | 14 day after application | | | | |
| Damage index (%) | Repeat 1 | 42.4 | 35.4 | 31.7 | 44.4 | 41.7 | 48.5 | 43.4 | 18.2 | 16.2 | 87.0 |
|  |  | 43.4 | 48.5 | 41.3 | 51.9 | 55.6 | 36.4 | 42.4 | 25.3 | 25.3 | 75.6 |
|  | Repeat 2 | 40.4 | 31.3 | 36.5 | 28.4 | 36.1 | 38.4 | 43.4 | 26.3 | 17.2 | 68.9 |
|  |  | 48.5 | 35.4 | 33.3 | 28.4 | 51.9 | 53.5 | 34.3 | 41.4 | 44.4 | 75.6 |
|  | Repeat 3 | 49.5 | 35.4 | 46.0 | 38.3 | 55.6 | 43.4 | 25.3 | 36.4 | 31.3 | 66.7 |
|  |  | 36.4 | 40.4 | 43.1 | 29.2 | 36.1 | 39.4 | 27.3 | 18.2 | 20.2 | 60.0 |
| Application time | | 3 day after application | | | | |  |  |  |  |  |
| Damage index (%) | Repeat 1 | 53.5 | 53.5 | 35.4 | 38.4 | 60.0 |  |  |  |  |  |
|  |  | 51.5 | 45.5 | 46.5 | 38.4 | 51.9 |  |  |  |  |  |
|  | Repeat 2 | 46.5 | 50.5 | 38.4 | 30.3 | 53.3 |  |  |  |  |  |
|  |  | 53.5 | 35.4 | 41.4 | 47.5 | 44.4 |  |  |  |  |  |
|  | Repeat 3 | 44.4 | 36.4 | 44.4 | 42.4 | 46.7 |  |  |  |  |  |
|  |  | 44.4 | 35.4 | 37.4 | 43.4 | 48.9 |  |  |  |  |  |
